# Development of polyvinyl alcohol-based carbon nano fiber sheet for thermal interface material

**DOI:** 10.1038/s41598-021-96691-z

**Published:** 2021-08-25

**Authors:** Jiangling Xiong, Siran Chen, Yongbum Choi, Kazuhiro Matsugi

**Affiliations:** 1 Mechanical Engineering Program, Graduate School of Advanced Science and Engineering, Hiroshima University, 1-4-1, Kagamiyama, Higashi-Hiroshima, Hiroshima 739-8527 Japan; 2 Department of Mechanical Materials Engineering, Hiroshima University, 1-4-1, Kagamiyama, Higashi-Hiroshima, Hiroshima 739-8527 Japan; 3 Graduate School of Advanced Science and Engineering, Hiroshima University, 1-4-1, Kagamiyama, Higashi-Hiroshima, Hiroshima 739-8527 Japan

**Keywords:** Polymers, Composites, Synthesis and processing

## Abstract

Polyvinyl alcohol (PVA)-based carbon nanofiber (CNF) sheets are fabricated as an innovative thermal interface material (TIM), which is a potential substitute for traditional TIMs. Five types of PVA-based CNF sheets were fabricated at different mass ratios of PVA:vapor-grown carbon fiber (VGCF) (1:0.100, 1:0.070, 1:0.050, 1:0.030, 1:0.025). The thickness of the PVA-based CNF sheets was 30–50 µm, which was controlled by the amount of VGCF. The microstructure of the CNF sheets indicated that VGCFs were arranged in random directions inside the sheet, and PVA was formed as a membrane between two VGCFs. However, many pores were found to exist between the VGCFs. The porosity of the PVA-based CNF sheets decreased from 25 to 13% upon decreasing the mass ratio of VGCF from 43.38 to 16.13%. The density and Shore hardness of all CNF sheets were 1.03–1.15 × 10^6^ g m^−3^ and 82.4–85.0 HS, respectively. The highest thermal conductivity, measured as the mass ratio of PVA:VGCF, was achieved at 1:0.05, with the in-plane thermal conductivity of the fabricated sheet being 14.3 W m^−1^ k^−1^.

## Introduction

The continuous development of the electronics industry demands for a rapid increase in the heat flux density of electronic components. Without efficient heat dissipation, malfunctions occur in junctions or modules of electronic components because of heat concentration^1^. Inefficient heat dissipation limits both performance and service life of electronic components. Thermal interface materials (TIMs), which are used to fill the air gaps between electronic components and heat sinks, reduce the thermal contact resistance and are, thus, considered important for improving the heat dissipation efficiency of electronic devices. To this end, many types of TIM materials have been explored, such as thermal pads, phase-change materials, graphite sheets, and metal sheets^2^.

Although grease is more commonly used as a TIM, it has a few disadvantages such as the difficulty with uniform spreading and the presence of voids. If grease is not appropriately spread, heat conduction from the heating element to the heat sink does not efficiently occur. Phase-change materials require a moderate contact pressure to bind surfaces together and their physical and chemical properties are unstable in processes wherein their long-term usage is required^3^. In recent years, the development of new TIMs has been accelerated through the use of carbon materials such as graphene, carbon nanotubes (CNTs), and carbon nanofibers (CNFs). These materials have been widely applied as fillers in TIMs because of their high thermal conductivity and excellent mechanical properties. Among these carbon materials, graphene TIMs exhibit highly anisotropic thermal conductivity because of their two-dimensional structure and high aspect ratio. For example, Jeon et al. developed cellulose-graphene-based TIM papers^4^. According to their studies, although the in-plane thermal conductivity of cellulose-graphene-based TIM paper can reach to 7.32 W m^−1^ K^−1^, the maximum through-plane thermal conductivity is only 0.14 W m^−1^ K^−1^. Huang et al. reported thermal conductivities of current graphene-based polymer nanocomposites. The thermal conductivities of most reported graphene-based polymer nanocomposites were under 10 W m^−1^ K^−1^ due to the high aspect ratio of graphene materials^5^. As for CNT TIMs, many studies have demonstrated that the properties of CNT TIMs cannot be completely exploited because of the presence of defects and high thickness, high interface thermal resistance, and low purity^6^. Hong et al. used single-walled carbon nanotubes (SWCNTs) and multi-walled carbon nanotubes (MWCNTs) to fabricate CNTs/polymethylmethacrylate (PMMA) composites. The maximum thermal conductivity of SWCNT/PMMA and MWCNT/PMMA composites are 2.43 W m^−1^ K^−1^ and 3.44 W m^−1^ K^−1^. They found that purity and defects of CNT have great influence on the thermal conductivity of CNT TIMs^7^. Moreover, commercialization of most reported TIMs is difficult considering the complexity in their large-scale manufacturing method and high cost. On the other hand, CNFs are discontinuous, highly graphitic, highly compatible with most polymer processing techniques, and they can be dispersed in an isotropic or anisotropic mode. As a type of CNFs, VGCF has excellent mechanical properties such as high thermal conductivity, high specific strength, and high corrosion resistance and is commercially available at a low cost. VGCF can be incorporated in a wide range of matrices, including thermoplastics and thermosets, for the formation of thin and flexible sheets. Therefore, as thermally conductive fillers, CNFs are preferred to other carbon materials. PVA, a resin, is expected to bind VGCFs and impart high flexibility to CNF sheets because of its high adhesion strength and high malleability.

The objective of this study is to fabricate PVA-based CNF sheets that exhibit high thermal conductivity and good workability and have a simple fabrication process. The microstructure of the PVA-based CNF sheets was determined through scanning electron microscopy (SEM), and their porosity was calculated by using an image analysis software. The thickness, density, and hardness of the PVA-based CNF sheets were also investigated. The in-plane and through-plane thermal conductivities of the PVA-based CNF sheets were measured using a periodic heating and infrared radiation thermometer method. In addition, a CNF sheet with high thermal conductivity was selected to perform constant temperature and humidity tests at 358 K and 85% RH, respectively, for 500 h.

## Materials and methods

**Figure 1:**
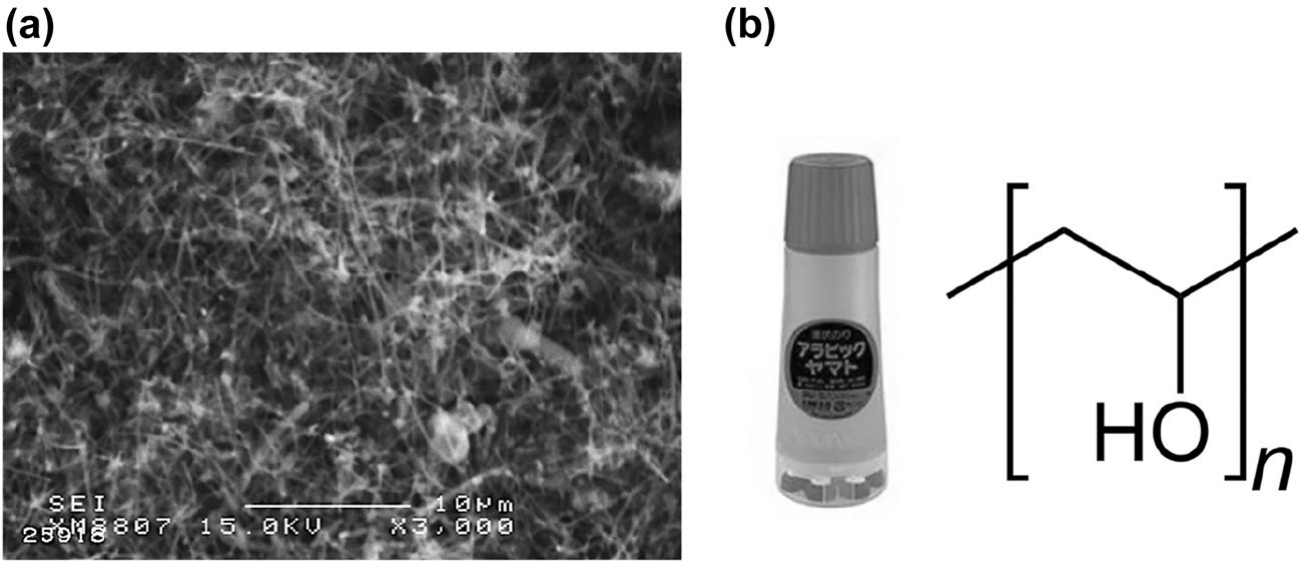
(**a**) SEM image of VGCFs, and (**b**) image and molecular structure of PVA.

**Figure 2:**
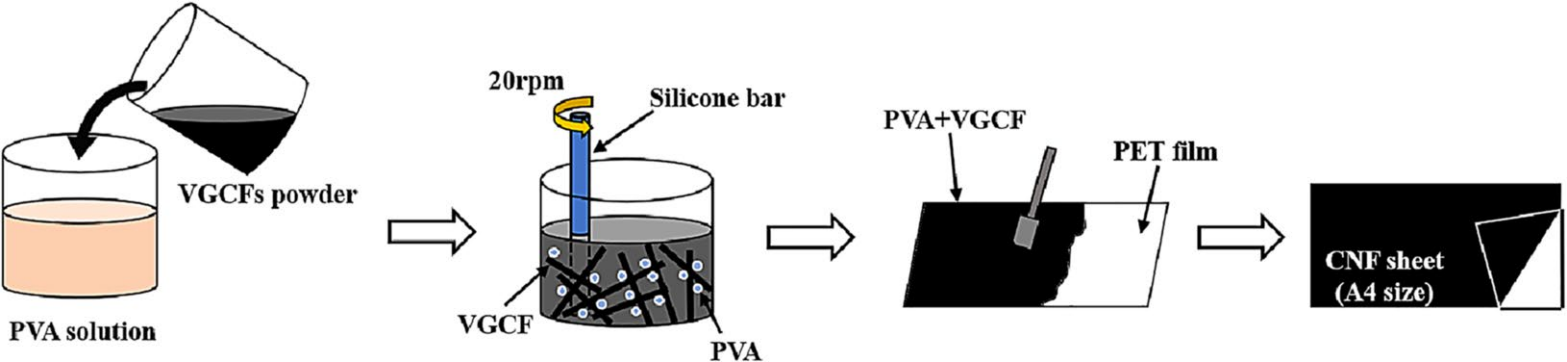
Schematic of the fabrication procedure of CNF sheet.

In this study, VGCF with a diameter of 150 nm and a length of 10–20 µm was used to achieve high thermal conductivity (Showa Denko Co., Ltd.). Moreover, PVA (Yamato Co., Ltd) with a concentration of 13 mass%, available as a liquid glue, was used as a binder. In A4 paper sheet production, the amount of PVA is typically constant, and the amount of VGCF to be added is varied. The scanning electron microscope (SEM) image of the as-received VGCF and the molecular structure of PVA are shown in Fig. 1. It should be emphasized that although the VGCF was readily aggregates, a dispersion medium to inhibit the aggregation was not used in this study. The VGCFs were utilized under as-received condition. The aggregation of VGCFs can contribute to provide a 3D net-like structure during the fabrication process, which is expected to improve the isotropic thermal conductivity of fabricated sheets. The manufacturing method for the CNF sheet is shown in Fig. 2. To obtain a TIM sheet with an isotropic thermal conductivity, the as-received VGCFs were poured into the PVA binder. After mixing it with a silicone bar at 20 rpm for 1 min, the VGCF–PVA mixture was spread on a polyethylene terephthalate (PET, (C_10_H_8_O_4_)_n_) film of A4 paper size. After drying the VGCF–PVA mixture at room temperature (RT) for 48 h, the CNF sheet was separated from the PET film. In this study, five types of CNF sheets were fabricated with different mass ratios of VGCF at a constant PVA amount, to investigate the effect of the amount of VGCF on thermal conductivity. The fabrication conditions of the CNF sheets are listed in Table 1. The porosity of each sheet was calculated through image analysis using Image Pro Plus 6.0, which used five random areas with a size of 250 μm^2^ from each CNF sheet. The density was calculated from the mass and volume of each fabricated sheet. The hardness of the CNF sheets was also tested using a Shore durometer. According to the ASTM D2240 procedure^8^, the CNF sheets with a total thickness of 5 mm were used for hardness measurement. The thermal properties of the CNF sheets were evaluated using a periodic heating and infrared radiation thermometer method with a thermophysical analyzer (Thermowave Analyzer TA). In addition, high-temperature and humidity tests were performed to evaluate the long-term performance of the CNF sheets at a temperature of 358 K and RH of 85% for 500 h, respectively.

**Table 1:** Fabrication conditions of CNF sheets.

Samples	PVA:VGCF (mass%)	VGCF (mass%)	VGCF (vol.%)
a	1:0.100	43.48	17.04
b	1:0.070	35.00	12.85
c	1:0.050	27.78	10.15
d	1:0.030	18.75	8.18
e	1:0.025	16.13	7.95

## Results and discussion

### Microstructures and porosity of CNF sheets

**Figure 3:**
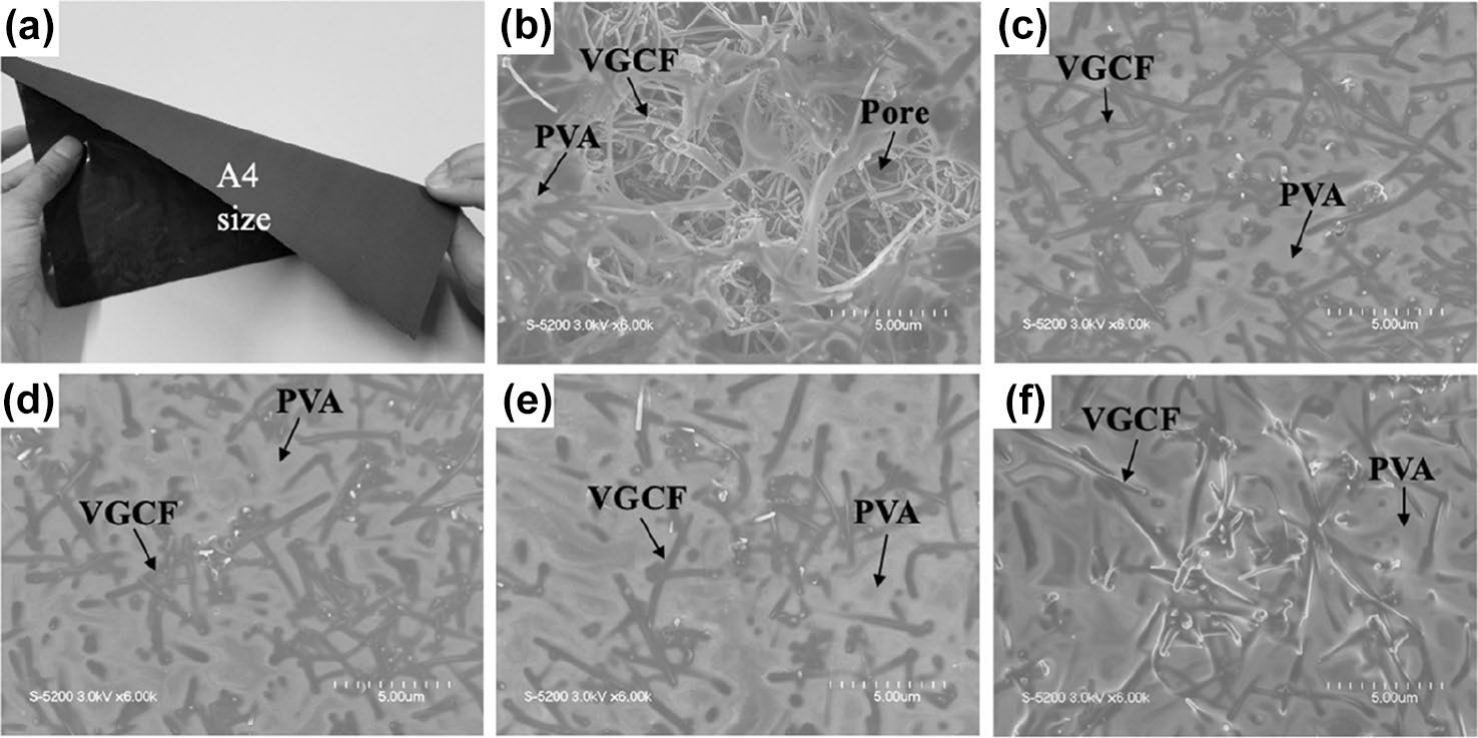
(**a**) The overall appearance of fabricated CNF sheet and SEM images of fabricated CNF sheets by different mass ratio of PVA:VGCF, (**b**) 1:0.100, (**c**) 1:0.070, (**d**) 1:0.050, (**e**) 1:0.030, (**f**) 1:0.025.

**Figure 4:**
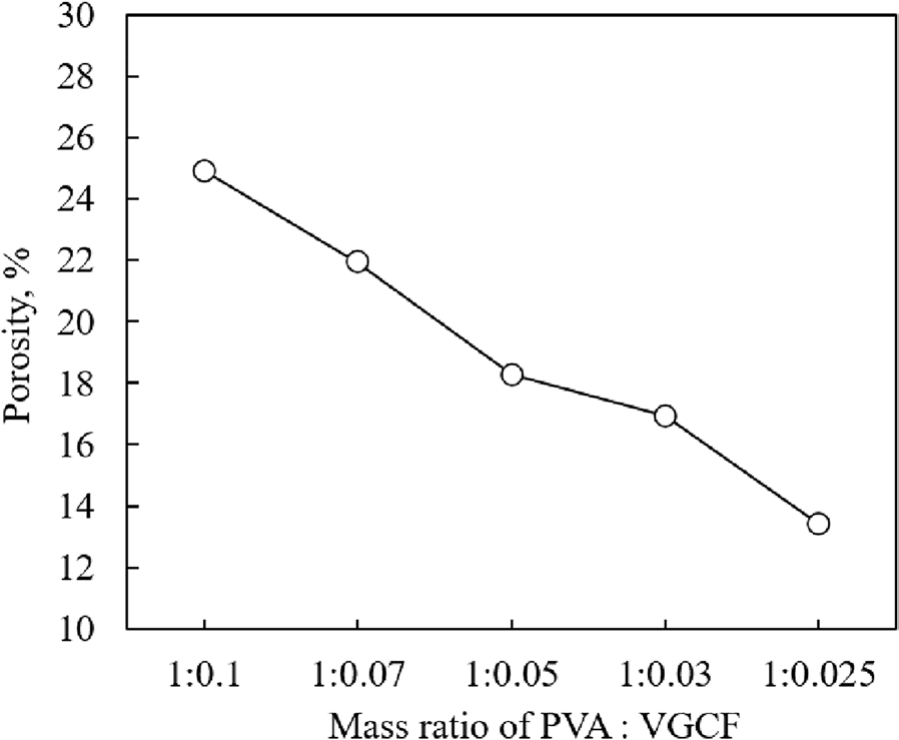
Effect of mass ratio of PVA and VGCF on porosity.

Figure 3 shows the photographs and SEM images of the CNF sheets. As shown in Fig. 3a, the fabricated CNF sheet was soft and flexible with a uniform surface. The structural flexible of TIM is one of great importance for practical application. Figure 3b–f show the SEM images of the microstructure of the fabricated CNF sheets with different mass ratios of PVA:VGCF (1:0.100, 1:0.070, 1:0.050, 1:0.030, and 1:0.025). All sheets showed that the VGCFs were arranged in random directions and connected with each other via PVA aggregates. However, in Fig. 3b, many pores were observed between the VGCFs. At the PVA:VGCF ratio of 1:0.100, due to the relatively low ratio of PVA, PVA can neither effectively infiltrate the VGCFs nor construct a uniform surface; thus, there were a large number of obvious pores exhibit in surface of CNF sheet. This ratio was not considered to be optimal. As shown in Fig. 3c–f, the decrease in the added VGCF amount resulted in the decrease in the number of pores between the VGCFs. The porosity of each CNF sheet was calculated using image analysis, and the results are shown in Fig. 4. The porosity of the CNF sheets decreased from 25 to 22%, 18%, 17%, and 13% by decreasing the amount of VGCFs, which is due to the number of intersections between the VGCFs. Decreasing the amount of VGCFs also decreased the number of intersections between VGCFs. Because of these intersections, the CNF sheets have a cellulose-like structure, through which the PVA infiltration between VGCFs is hindered during the mixing process, thereby leading to pore formation in the fabricated sheet. Therefore, decreasing the amount of VGCFs will contribute to the formation of the CNF sheet with low porosity.

### Thickness, density and hardness

**Figure 5:**
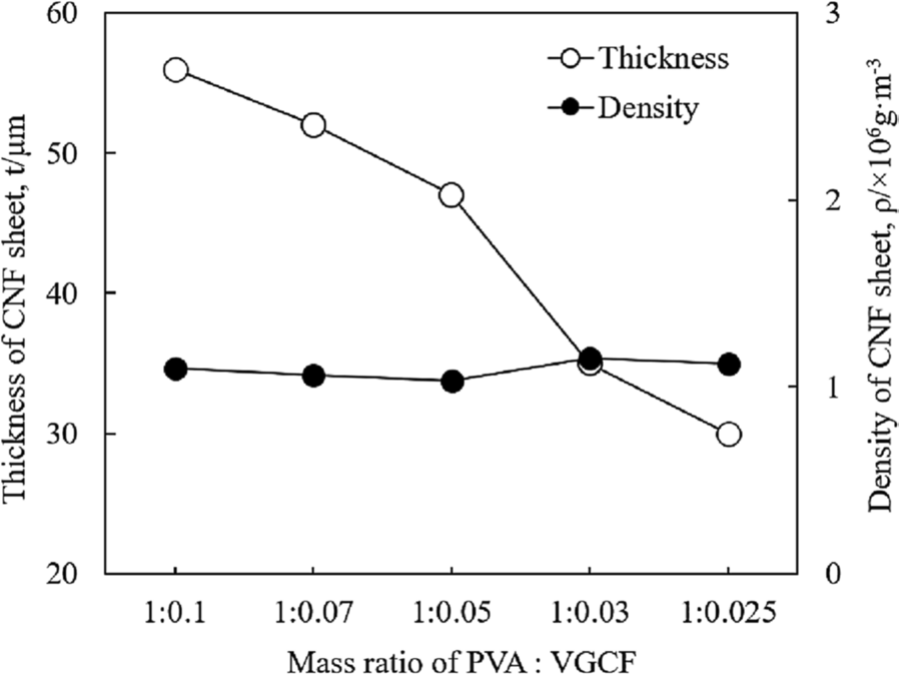
Effect of mass ratio of PVA and VGCF on the thickness and density of CNF sheets.

**Figure 6:**
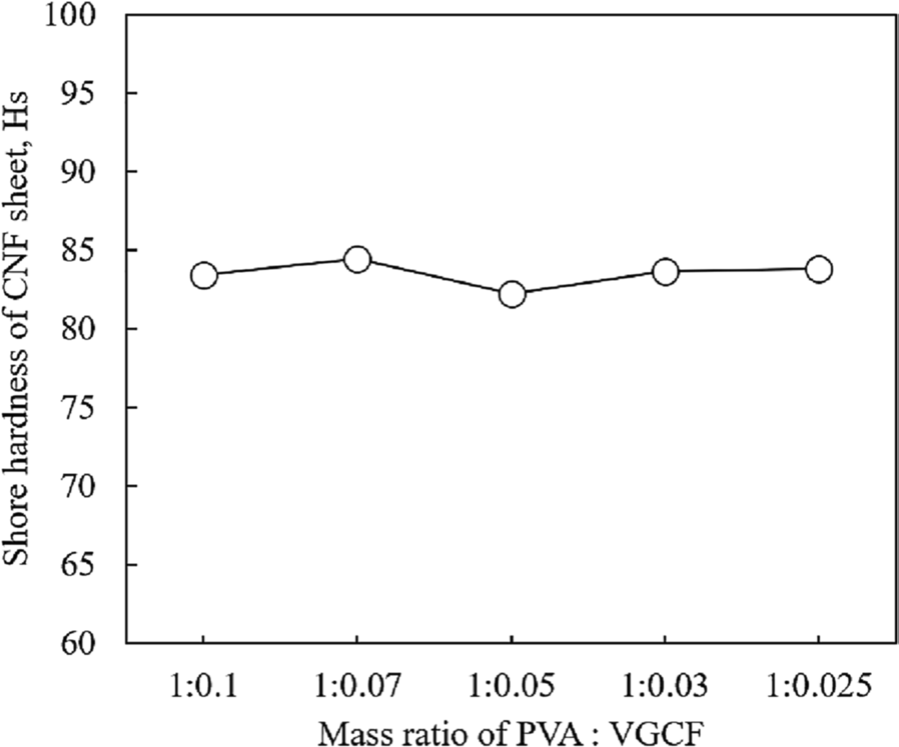
Shore hardness of CNF sheets by different mass ratio of PVA and VGCF.

To evaluate the possibility of the fabricated CNF sheets for practical application of TIM, thickness, density and Shore hardness of CNF sheets were measured. Figure 5 shows the relationship between thickness and density based on the mass ratio of PVA:VGCF. The thickness of each CNF sheet was 56, 52, 47, 35, and 30 µm, and the fabricated CNF sheets become thicker with decreasing amounts of VGCFs. Considering the existence of a flat and smooth surface between the semiconductor and heat sink, it is effective to maintain the thickness of the TIM to ≤ 0.5 mm. In addition, the air gaps between the semiconductor and heat sink could not be effectively filled in wafer-thin TIMs. Therefore, the thicknesses of CNF sheet b (PVA:VGCF = 1:0.070) and sheet c (PVA:VGCF = 1:0.050), which are 52 and 47 µm, respectively, were considered to be optimal for TIM. The density of each CNF sheet was calculated using mass and volume and its values were 1.10 × 10^6^, 1.06 × 10^6^, 1.03 × 10^6^, 1.15 × 10^6^, and 1.12 × 10^6^ g m^−3^. The density results showed the lightweight property of CNF sheets compared to commercially available TIMs (1.44–3.6 × 10^6^ g m^−3^)^9^. It was also found that the CNF sheets maintained a relatively constant density although they were fabricated using different mass ratios of PVA and VGCF. In the hardness test, according to the ASTM D2240 procedure, each type of CNF sheet was superimposed onto the same thickness sheet (5 mm), and its Shore hardness was measured. The results are presented in Fig. 6. The hardness of each CNF sheet was 83.4, 84.4, 82.2, 83.6 and 83.8 HS. There was no obvious difference between each CNF sheet in terms of Shore hardness, and a constant hardness was successfully obtained. It is interesting that CNF sheets have similar Shore hardness to that of commercial soft lining material^10^, which indicated the great softness and flexibility of CNF sheets for practical application of TIM.

### Thermal conductivity

**Figure 7:**
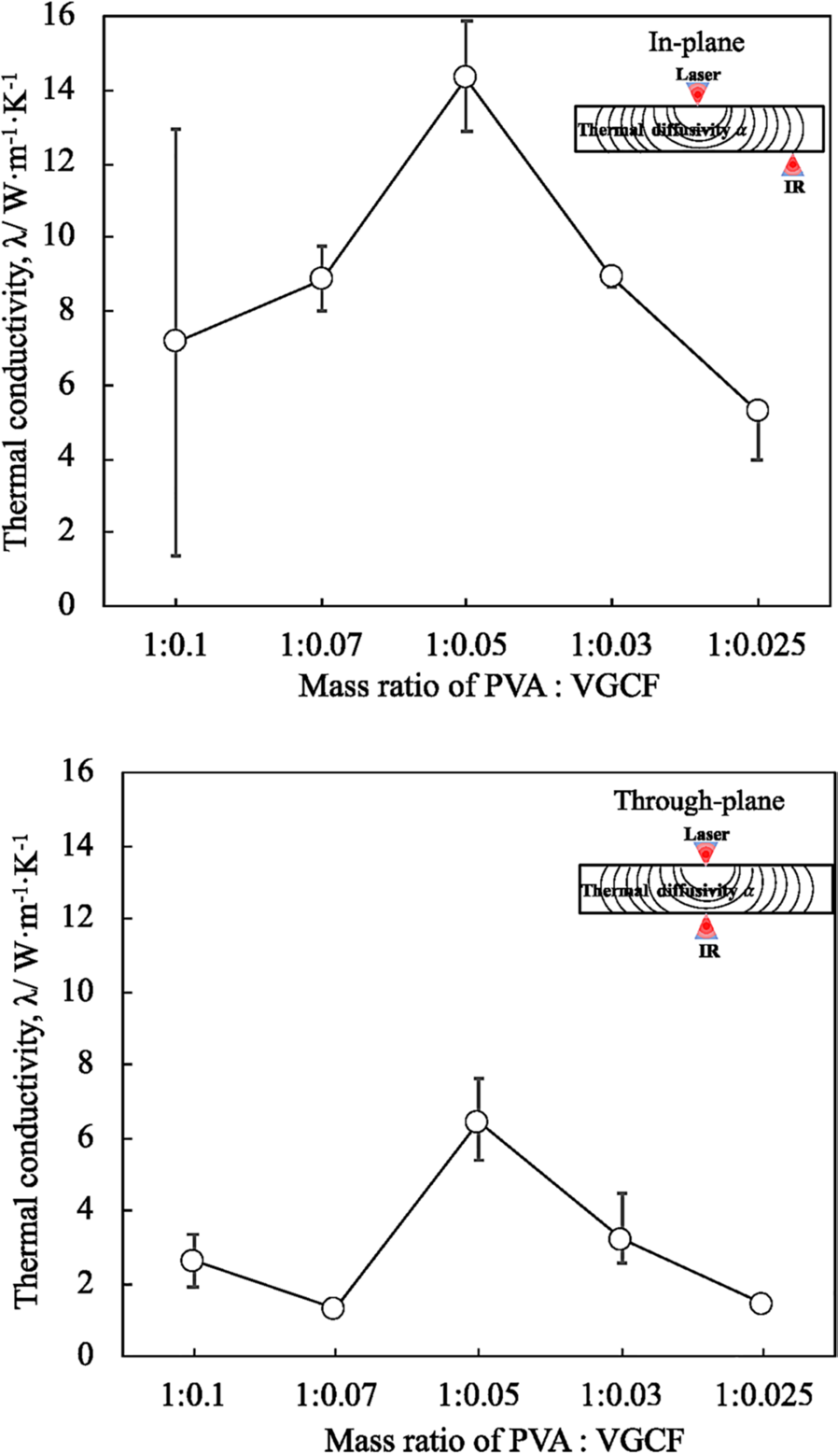
Effect of mass ratio of PVA and VGCF on in-plane and through-plane thermal conductivity.

Figure 7 shows the effect of the PVA:VGCF mass ratio on the in-plane and through-plane thermal conductivities of the CNF sheet. The pure PVA resin has a low thermal conductivity of approximately 0.28 W m^−1^ K^−1^, and the VGCF used in this study has a high thermal conductivity of 1200.00 W m^−1^ K^−1^ at RT^11^. As shown in the figure, the CNF sheets were fabricated by varying the amount of VGCFs: 1:0.100 (43.48 mass%), 1:0.070 (35.00 mass%), 1:0.050 (27.78 mass%), 1:0.030 (18.75 mass%), 1:0.025 (16.13 mass%); the respective in-plane thermal conductivities were 12.94, 8.87, 14.30, 8.90, and 5.29 W m^−1^ K^−1^ and through-plane thermal conductivities were 3.40, 1.34, 6.44, 3.25, and 1.51 W m^−1^ K^−1^. Among all specimens, CNF sheet c exhibited the maximum in-plane and through-plane thermal conductivities of 14.30 and 6.44 W m^−1^ K^−1^, respectively. Moreover, both these conductivities showed a non-linear relationship with the VGCF amount, suggesting that the thermal conductivity was not only influenced by the amount of VGCFs. As mentioned in “Microstructures and porosity of CNF sheets”, the porosity of the CNF sheet would increase by increasing the amount of VGCF. The pores in the CNF sheet were close to that of air gaps, which decreased the thermal properties and density. High porosity led to lower thermal conductivities of CNF sheet a and b. Moreover, overloading of CNFs may also result in a deterioration of mechanical properties of CNF sheet. Therefore, minimizing the amount of CNFs while keeping high thermal properties is preferable for sheet fabrication^12^. In addition, there was a large thermal conductivity error of CNF sheet a in Fig. 7, it is also caused by the high porosity. Firstly, the thermophysical properties of the CNF sheets are measured using periodical heating radiation-temperature measuring method in this study, the laser diameter is 100–150 μm, which means the result is easily affected by the surface condition and roughness of sample. Meanwhile, due to the high porosity of CNF sheet a, a large number of pores can readily structure an undulating surface. Considering the thickness of CNF sheet a is only 56 μm, some large pores shown in Fig. 3b could also lead to an obvious reduction of thickness in this area. The undulating surface and uneven thickness of CNF sheet a have strong influence on heat radiation and calculation of thermal conductivity. Therefore, there was a large thermal conductivity error of CNF sheet a. As for CNF sheet d and e, the low thermal conductivity is caused by the low VGCF amount and the irregularity of the surface. Therefore, the maximum thermal conductivities were showed in CNF sheet c with an optimal amount of VGCF (PVA:VGCF = 1:0.05). Table 2 lists previously reported thermal conductivity of other carbon-based thermally conductive materials. As shown in this table, the through-plane thermal conductivity of CNF sheet in this study is significantly higher than those of previous studies. In-plane thermal conductivity of CNF sheet is still higher than the most of reported thermally conductive materials. Moreover, commercialization of most reported materials is difficult considering the complexity in their large-scale manufacturing method and high cost. By using a simple and cost-effective process, the integration of high thermal conductivity, lightweight and super flexibility makes the CNF sheets highly promising for heat management of advanced electronic device.

**Table 2:** Comparison of thermal conductivity of our CNF sheet with other carbon-based thermally conductive materials.

Material	Matrix	Fraction	Thickness (μm)	In-plane Thermal conductivity (W m^−1^ K^−1^)	Through-plane thermal conductivity (W m^−1^ K^−1^)	References
CNF	PVA	10.15 vol%	47	14.30	6.44	This study
3D-Carbon fibers network	Epoxy	13 vol%	Unknown	1.70	2.84	^13^
CNT	Epoxy	16.7 vol%	Unknown	1.00	4.87	^14^
Graphene paper	Oriental tradition paper	Unknown	190	7.32	0.14	^4^
Graphene aerogel	Epoxy	0.92 vol%	Unknown	0.63	2.13	^15^
Templated graphene framework	Epoxy	8.3 wt%	Unknown	8.80	2.00	^16^
Graphene nanoplatelet	Nature rubber	27.48 vol%	71	39.27	1.50	^17^

### High temperature and humidity test

**Figure 8:**
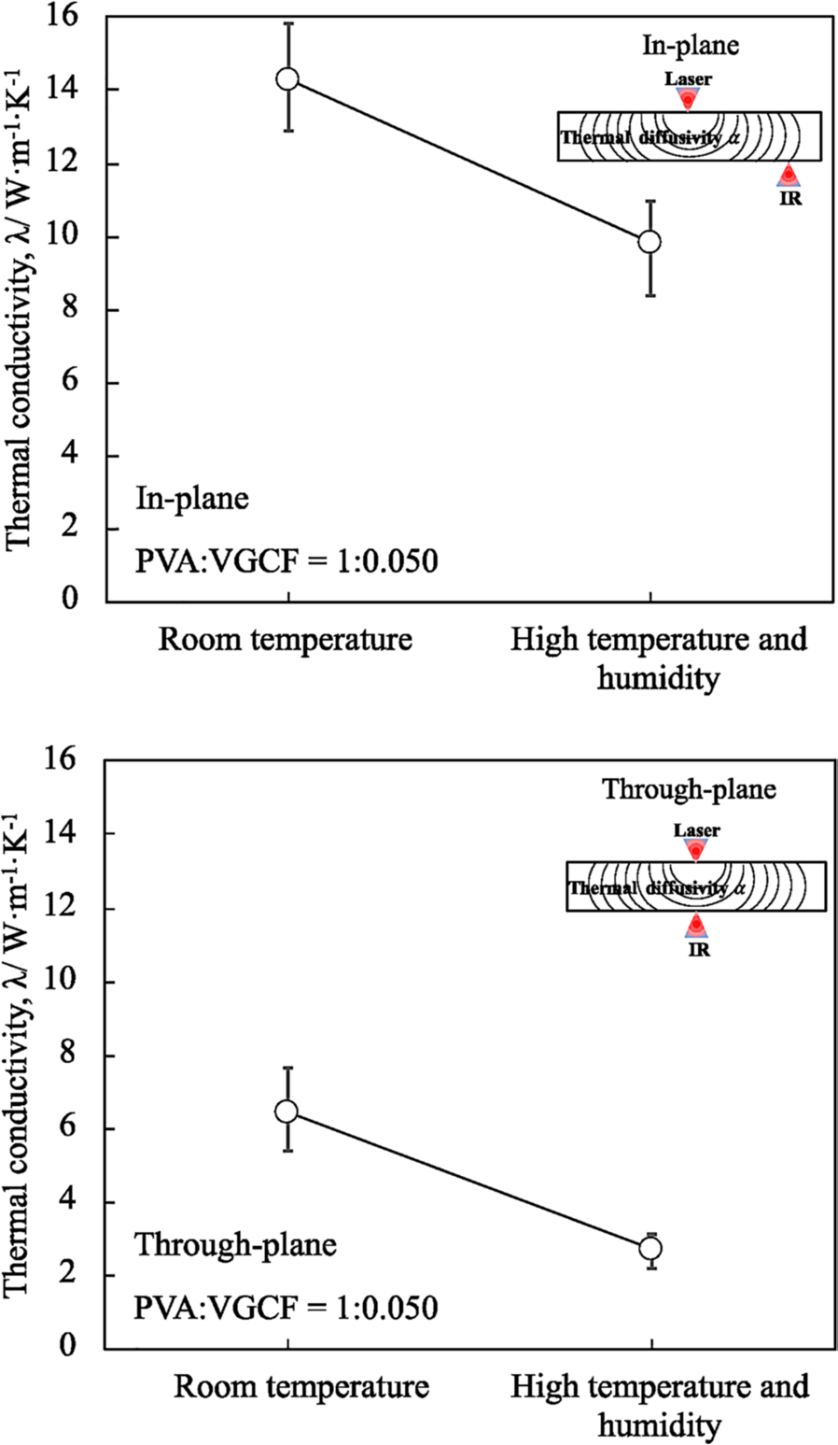
In-plane and through-plane thermal conductivity of specimens before and after the high temperature and humidity test.

In this study, the CNF sheet (PVA:VGCF = 1:0.050) with the highest thermal conductivity was cut into four specimens with a size of 20 × 20 mm for the high temperature and humidity tests. Each specimen was held at 358 K and 85% RH for 500 h. After the high temperature and humidity tests, the average in-plane and through-plane thermal conductivities of the four specimens were measured. As shown in Fig. 8, prior to testing, the in-plane thermal conductivity was high at 14.30 W m^−1^ K^−1^; however, after testing, the average in-plane thermal conductivity of the four specimens decreased to 9.85 W m^−1^ K^−1^, with a reduction of 31%. Similarly, prior to testing, the through-plane thermal conductivity was 6.44 W m^−1^ K^−1^, which decreased to 2.71 W m^−1^ K^−1^, with a reduction of ~ 57% after testing. It is considered that the reduction of thermal conductivity is caused by the hydrophilic property of PVA. The water molecules in the environment would be absorbed into CNF sheet by PVA. The absorbed water in PVA not only disrupts hydrogen bonding, but also contributes more free volume and lubrication. The PVA segments in CNF sheet become readily mobile and rapidly responding to the load change^18^, which may lead to a movement of PVA from surface to center part of CNF sheet. A large number of pores would be generated in surface of CNF sheet. Therefore, absorbed water in PVA degraded the thermal properties and increased the porosity of the CNF sheet.

## Conclusions

In this study, an innovative TIM based on a composite of PVA and VGCF was successfully fabricated by a simple and cost-effective process. Five types of CNF sheets were fabricated by varying the amount of VGCF, and their in-plane and through-plane thermal conductivities were measured. The mechanical properties of each CNF sheet were also tested. The main findings of this study are listed below:

Microstructure observation revealed that the VGCFs were arranged in random directions and interconnected via PVA aggregates. Numerous pores existed between the VGCFs due to water evaporation. The porosity of the CNF sheets decreased by decreasing the VGCF amount, which is attributed to the fewer intersections between the VGCFs.

The thickness of the CNF sheets was between 30 and 56 µm and increased with increasing the VGCF amount. Moreover, the CNF sheets did not exhibit considerably different density and hardness: the average density of the CNF sheet was ~ 1.00 × 10^6^ g m^3^, and the Shore hardness of the CNF sheets with a total thickness of 5 mm was between 82 and 85 HS. It is indicated CNF sheets was fabricated with suitable thickness, lightweight property and great flexibility for practical application of TIM.

The highest value of thermal conductivity was obtained for the CNF sheet fabricated at the PVA:VGCF mass ratio of 1:0.050, with the in-plane and through-plane thermal conductivities being 14.30 and 6.44 W m^−1^ K^−1^, respectively. Reducing the VGCF amount decreased the thermal conductivity. Furthermore, an excessive increase in the VGCF amount increased the number of pores in the CNF sheet, which led to thermal conductivity reduction.

The in-plane and through-plane thermal conductivities were significantly reduced after the high-temperature and humidity tests. It is considered that due to the hydrophilic property of PVA, the water molecules in the environment would be absorbed into CNF sheet by PVA, which led to high porosity and thermal conductivity reduction.

## Data Availability

The datasets generated and analysed during the current study are available from the corresponding author on reasonable request.
